# Osthole induces necroptosis via ROS overproduction in glioma cells

**DOI:** 10.1002/2211-5463.13069

**Published:** 2021-01-19

**Authors:** Mengjie Huangfu, Riming Wei, Juan Wang, Jianli Qin, Dan Yu, Xiao Guan, Xumei Li, Minglei Fu, Haiping Liu, Xu Chen

**Affiliations:** ^1^ College of Pharmacy Guilin Medical University China; ^2^ Institute of Biotechnology Guilin Medical University China; ^3^ School of Basic Medical Sciences Guilin Medical University China; ^4^ Xiangya Hospital Central South University Changsha China; ^5^ The Second Affiliated Hospital of Guilin Medical University China; ^6^ Science and Technology Department Guilin Medical University China

**Keywords:** cell necroptosis, glioma, osthole, ROS production

## Abstract

Glioma is a common primary malignant tumor that has a poor prognosis and often develops drug resistance. The coumarin derivative osthole has previously been reported to induce cancer cell apoptosis. Recently, we found that it could also trigger glioma cell necroptosis, a type of cell death that is usually accompanied with reactive oxygen species (ROS) production. However, the relationship between ROS production and necroptosis induced by osthole has not been fully elucidated. In this study, we found that osthole could induce necroptosis of glioma cell lines U87 and C6; such cell death was distinct from apoptosis induced by MG‐132. Expression of necroptosis inhibitor caspase‐8 was decreased, and levels of necroptosis proteins receptor‐interacting protein 1 (RIP1), RIP3 and mixed lineage kinase domain‐like protein were increased in U87 and C6 cells after treatment with osthole, whereas levels of apoptosis‐related proteins caspase‐3, caspase‐7, and caspase‐9 were not increased. Lactate dehydrogenase release and flow cytometry assays confirmed that cell death induced by osthole was primarily necrosis. In addition, necroptosis induced by osthole was accompanied by excessive production of ROS, as observed for other necroptosis‐inducing reagents. Pretreatment with the RIP1 inhibitor necrostatin‐1 attenuated both osthole‐induced necroptosis and the production of ROS in U87 cells. Furthermore, the ROS inhibitor *N*‐acetylcysteine decreased osthole‐induced necroptosis and growth inhibition. Overall, these findings suggest that osthole induces necroptosis of glioma cells via ROS production and thus may have potential for development into a therapeutic drug for glioma therapy.

## Graphical abstract

Osthole inhibits the expression of caspase‐8, activates the receptor‐interacting protein 1 (RIP) 1/RIP3/mixed lineage kinase domain‐like protein pathway and triggers reactive oxygen species (ROS) production, which causes glioma cell necroptosis. Moreover, treatment with a RIP1 inhibitor decreased ROS production and RIP1 expression, which suggests that a positive feedback regulation loop of ROS and RIP1 may exist.

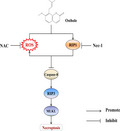

**Abbreviations**
DCFH‐DA: 2′, 7′‐dichloro‐hydrofluorescein diacetate
DDW: double‐distilled water
LDH: lactate dehydrogenase
MFI: mean fluorescence intensity
MLKL: mixed lineage kinase domain‐like protein
MMP: mitochondrial membrane potential
MTT: 3‐(4,5‐dimethylthiazol‐2‐yl)‐2,5‐diphenyl‐tetrazolium bromide
NAC: *N*‐acetylcysteine
Nec‐1: necrostatin‐1
PI: propidium iodide
RIP1: receptor‐interacting protein 1
RIP3: receptor‐interacting protein 3
ROS: reactive oxygen species
SD: standard deviation

Glioma is the most common central nervous system tumor in adults and children [1, 2], and glioblastoma is the most malignant glioma ranked grade Ⅳ [3]. The average incidence of glioblastoma is 3.2/100 000 population, and the 5‐year survival rate is about 6.8% [4]. Although the occurrence of glioblastoma is relatively rare, it will cause serious consequences once it occurs [5]. The prognosis of glioblastoma patients is extremely poor, and its median survival time is about 15 months [6]. Chemotherapy and radiotherapy were used to treat gliomas, but gliomas are resistant to these therapeutics [7]. However, recent studies have found that inducing cell necroptosis can kill various cancer cells, including gliomas [8], breast cancer [9], and osteosarcoma [10]. Thus, inducing necroptosis may become an effective strategy to heal glioma.

Necroptosis is a newly found programmed cell death (PCD). It is regulated by a caspase‐independent pathway and has morphological features of necrosis [11, 12]. Receptor‐interacting proteins 1 and 3 (RIP1 and RIP3), the two critical kinases, are essential for necroptosis. Activated RIP1 combines with RIP3 to form a necrosome complex, and then recruits and activates mixed lineage kinase domain‐like protein (MLKL) [13, 14]. Activated MLKL oligomerizes and targets the cell membrane to promote the formation of pore structures, leading to necroptosis [15]. Increasing evidence indicated that reactive oxygen species (ROS) can activate various cell death, including necroptosis [16]. It has been reported that ROS promotes necroptosis by a positive feedback mechanism in osteoblastic cells, while ROS inhibitor *N*‐acetylcysteine (NAC) attenuates the up‐regulation of RIPK1, RIPK3 and MLKL, and the RIP1 inhibitor necrostatin‐1 (Nec‐1) reduced ROS generation [17]. Moreover, it has been reported that shikonin induces glioma cells necroptosis mediated by ROS [8]. Thus, ROS plays an important role in the induction of necroptosis.

Osthole is a natural coumarin derivative isolated from the *Cnidium monnieri* (L.) *Cusson*. Numerous studies demonstrated that antitumor bioactivity is an important pharmacological activity of osthole [18, 19, 20]. However, most studies focus on its antitumor mechanism of apoptosis induction instead of necrosis induction [21, 22]. Moreover, it has been proved that osthole could increase the level of ROS in cancer cells [23], whereas whether osthole‐induced necroptosis is related to ROS production is still unclear. So, in this study, we tested whether osthole‐induced cell death is related to ROS‐mediated necroptosis in U87 and C6 cells.

## Materials and methods

### Reagents

Osthole (purity > 98%) was purchased from Meilunbio (Dalian, China). MG‐132 and Nec‐1 were purchased from MedChemExpress (Shanghai, China). NAC and JC‐1 were purchased from Beyotime (Shanghai, China). DMSO and 2′, 7′‐dichloro‐hydrofluorescein diacetate (DCFH‐DA) were purchased from Sigma‐Aldrich (St. Louis, MO, USA). Anti‐rabbit IgG, anti‐mouse IgG, and antibodies against β‐actin were purchased from ZSGB‐BIO (Beijing, China). The anti‐RIP1 IgG, anti‐caspase‐3 IgG, anti‐caspase‐7 IgG and anti‐caspase‐9 IgG were purchased from Cell Signaling Technology (Danvers, MA, USA). Anti‐RIP3 IgG, anti‐MLKL IgG and anti‐caspase‐8 IgG were purchased from Abcam (Cambridge, MA, USA).

### Cell culture

The human U87 and rat C6 glioma cell lines (derived from glioblastoma) were obtained from Conservation Genetics CAS Kunming Cell Bank (Kunming, China). The HEB normal brain glial cell line was obtained from Shanghai Institute of Biochemistry and Cell Biology (Shanghai, China). The cells were cultured in Dulbecco's modified Eagle's medium (Gibco, Grand Island, NY, USA) containing 10% FBS (Gibco, Auckland, New Zealand), 100 U·mL^−1^ penicillin and 100 μg·mL streptomycin. The cells were kept in a humidified condition with 5% CO_2_ at 37 °C. Then the cells in the logarithmic growth phase were selected for the following experiments. The cells were pretreated with or without ROS inhibitor NAC (2 μm) and RIP1 inhibitor Nec‐1 (100 μm) for 1 h before osthole treatment. Apoptosis inducer MG‐132 (20 μm) treated cells for 18 h.

### Cell viability assay

Cells were seeded in 96‐well plates at 3 × 10^3^ cells per well and incubated at 37 °C, 5% CO_2_ for 24 h. After that, cells were treated with osthole at indicated concentrations for 24, 48 and 72 h. After treatment, 20 μL 3‐(4,5‐dimethylthiazol‐2‐yl)‐2,5‐diphenyl‐tetrazolium bromide (MTT) was added to each well, and cells continued to incubate for 4 h at 37 °C. Then the supernatant was aspirated, and 150 μL DMSO was added to dissolve the formed formazan crystals. Finally, the absorbance (*A*) was measured at 490 nm using a multimode reader (TECAN, Männedorf, Switzerland).

### Detection of necrosis by flow cytometry

We determined the way of cell death by using flow cytometry (Becton Dickinson, San Jose, CA, USA). In brief, cells were seeded in six‐well plates at 2 × 10^5^ cells per well and incubated for 24 h. After treatment with the indicated osthole for 18 h, the cells were harvested and washed with PBS. Then the cells were suspended in 300 μL binding buffer, added to 5 μL Annexin V–FITC and incubated at room temperature for 20 min in the dark. After that, 5 μL propidium iodide (PI) was added to the cells 5 min before the test. Two hundred microliters binding buffer was added before the cells were tested by flow cytometer (Becton Dickinson, San Jose, CA, USA). We collected 1 × 10^4^ cells per sample and analyzed cell necrosis rate by flowjo (Becton Dickinson, San Jose, CA, USA).

### Lactate dehydrogenase release assay

The lactate dehydrogenase (LDH) release was detected by the LDH assay kit (Nanjing Jiancheng Bioengineering Institute, Nanjing, China). In brief, 4 × 10^5^ cells per well were seeded in six‐well plates for 24 h, and indicated concentrations of osthole were intervened in 18 h. Then the cell culture medium was collected to detect the LDH release. Blank group (A), standard group (B), measurement group (C) and control group (D) were set. Group A was added to 25 μL double‐distilled water (DDW), group B was added to 5 μL DDW and 20 pyruvate standard solution (0.2 μm), group C was added to 20 μL medium and coenzyme I, and group D was added to 5 μL DDW and 20 μL medium. These samples were incubated at 37 °C for 15 min. Then, 25 μL 2, 4‐dinitrophenylhydrazine was added into samples at 37 °C for 15 min. Finally, 250 μL NaOH solution was added, and detected at 450 nm. Concentrations of LDH were calculated according to the following formula:

$\text{LDH}\;\left(U/L\right)=\frac{{\text{OD}}_{C}‐{\text{OD}}_{D}}{{\text{OD}}_{B}‐{\text{OD}}_{A}}\times \text{concentration of pyruvate standard solution}\;\left(0.2\;\mu M\right)\times 1000$.

Results were normalized to the control group.

### Mitochondrial membrane potential detection

Cell culture and osthole treatment are consistent with LDH detection. After that, cells were collected and suspended with PBS. JC‐1 fluorescent probe (0.2 μm) was added and incubated at room temperature for 20 min in the dark. After that, samples were washed with PBS, and the changes of mitochondrial membrane potential (MMP) were determined by flow cytometry. We collected 1 × 10^4^ cells per sample, and the mean fluorescence intensity (MFI) was calculated using flowjo.

### Detection of intracellular ROS

Cell culture was consistent with LDH detection. After treatment with osthole for 4 h, DCFH‐DA (2 μm) was added to the cell culture medium and incubated at 37 °C for 30 min in the dark. After staining, the cells were washed with PBS, and 1 × 10^4^ cells per sample were collected. Detection was performed on a flow cytometer, and the MFI was calculated using flowjo.

### Western blotting analysis

Cells (1 × 10^6^) were seeded in 100‐mm Petri dishes, cultured for 24 h and treated with indicated concentrations of osthole for 18 h. Then cells were collected, washed twice with PBS, lysed with radioimmunoprecipitation assay lysate buffer (Beyotime) for 30 min on ice, and centrifuged at 13 362 *g*. for 10 min. The supernatant was aspirated, and the protein concentrations were measured using a bicinchoninic acid kit (Beyotime). The protein was separated by SDS/PAGE electrophoresis and transferred to the nitrocellulose membranes. These membranes were blocked with 5% skim milk for 2 h and washed three times with PBST for 10 min each time. Then membranes were incubated with anti‐RIP1 (1 : 2000), anti‐RIP3 (1 : 4000), anti‐MLKL (1 : 4000) or anti‐β‐actin (1 : 4000) at 4 °C overnight. After that, membranes were washed with PBST three times for 10 min and incubated with horseradish peroxidase‐conjugated goat anti‐rabbit (1 : 4000) or anti‐mouse (1 : 4000) IgG diluted with 5% skim milk for 1 h at room temperature. Protein bands were detected by ECL Western Blotting Substrates (Bio‐Rad, Hercules, CA, USA), and gray values were analyzed by image j (National Institutes of Health, Bethesda, MD, USA).

### Statistical analysis

Data were analyzed by graphpad prism 8 software (GraphPad Software Inc., San Diego, CA, USA), and results were expressed as mean ± standard deviation (SD). Student's *t*‐test and one‐way ANOVA with the *post hoc* Bonferroni's test were conducted to analyze the statistical significance between different groups. *P* < 0.05 was considered statistically significant.

## Results

### Osthole inhibited the viability and induced cell necrosis in U87 and C6 cells

**Fig. 1:**
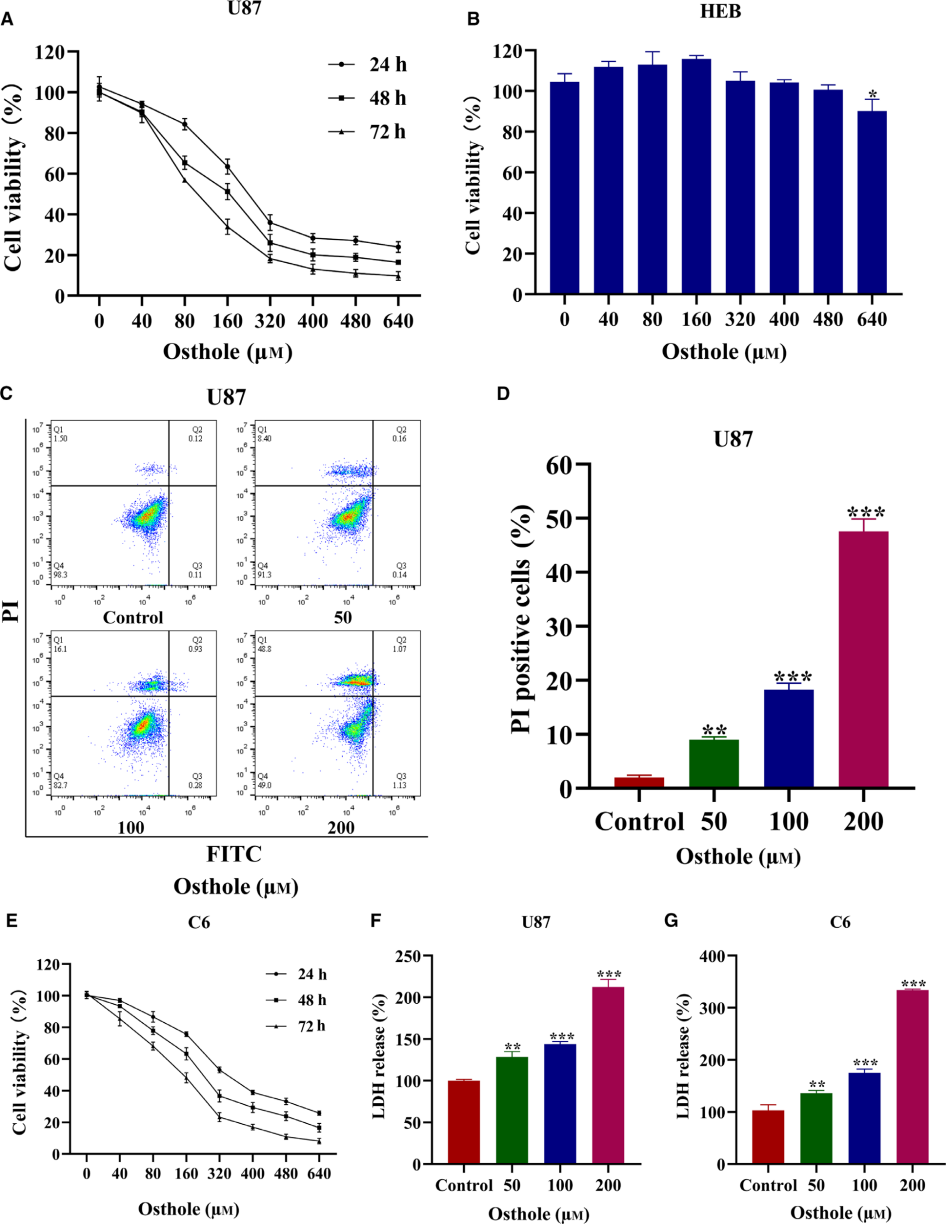
Osthole reduced cell viability and induced cell death in U87 and C6 cells. (A) U87 cells were treated with osthole at indicated concentrations for 24, 48 and 72 h. Cell viability was detected by MTT assay. (B) HEB cells were treated with osthole at indicated concentrations for 18 h. Cell viability was detected by MTT assay. (C) After treatment with osthole for 18 h, Annexin Ⅴ‐FITC/PI double staining was used to detect the form of cell death by flow cytometry. (D) Count of PI‐positive cells in osthole‐treated U87 cells. (E) C6 cells were treated with osthole at indicated concentrations for 24, 48 and 72 h. Cell viability was detected by MTT assay. (F, G) LDH release in U87 and C6 cells was tested after treatment with indicated osthole concentrations for 18 h. Data were presented as mean ± SD (*n* = 3). ANOVA with Bonferroni's *post hoc* test was used to test differences between multiple groups. *P < 0.05, **P < 0.01, ***P < 0.001.

To determine the cytotoxicity of osthole in U87 cells, we used the MTT assay. Compared with the control group, the cell viability gradually decreased with the increase of osthole concentration and incubation time. Our results revealed that osthole inhibited the viability in a dose‐ and time‐dependent manner (Fig. 1A). Furthermore, we found that osthole had no effect on normal human brain glial HEB cells until its concentration increased to 640 μm (Fig. 1B). Next, we used Annexin Ⅴ–FITC/PI dual staining to identify whether osthole induced cell death, and we found that the living cells in the osthole‐treated group were reduced significantly as the drug concentrations increased. In particular, the death cells nearly existed only in the necrosis group (Q1 quadrant), and PI‐positive cells were significantly increased (Fig. 1C,D). These results suggested that osthole inhibited cell viability and induced cell necrosis in U87 cells. Osthole mainly induced necrosis in U87 cells, which increased our interest. Then we verified necrosis induction of osthole in the C6 glioma cell line. We found that osthole also inhibited the viability in a dose‐ and time‐dependent manner in C6 cells (Fig. 1E). Moreover, we detected the LDH release in osthole‐treated U87 and C6 cells, which indicated plasma membrane rupture. Compared with the control group, LDH release increase was detected in osthole intervened cells (Fig. 1F,G). Because 200 μm osthole induced more LDH release than the other groups, this concentration was chosen. As the incubation time increased, the LDH release also increased significantly by 200 μm osthole (Fig. S1A,B). However, there was no significant difference in LDH release between 18 and 24 h. Thus, we chose 18 h as the incubation time.

### Osthole induced necroptosis in glioma cells

**Fig. 2:**
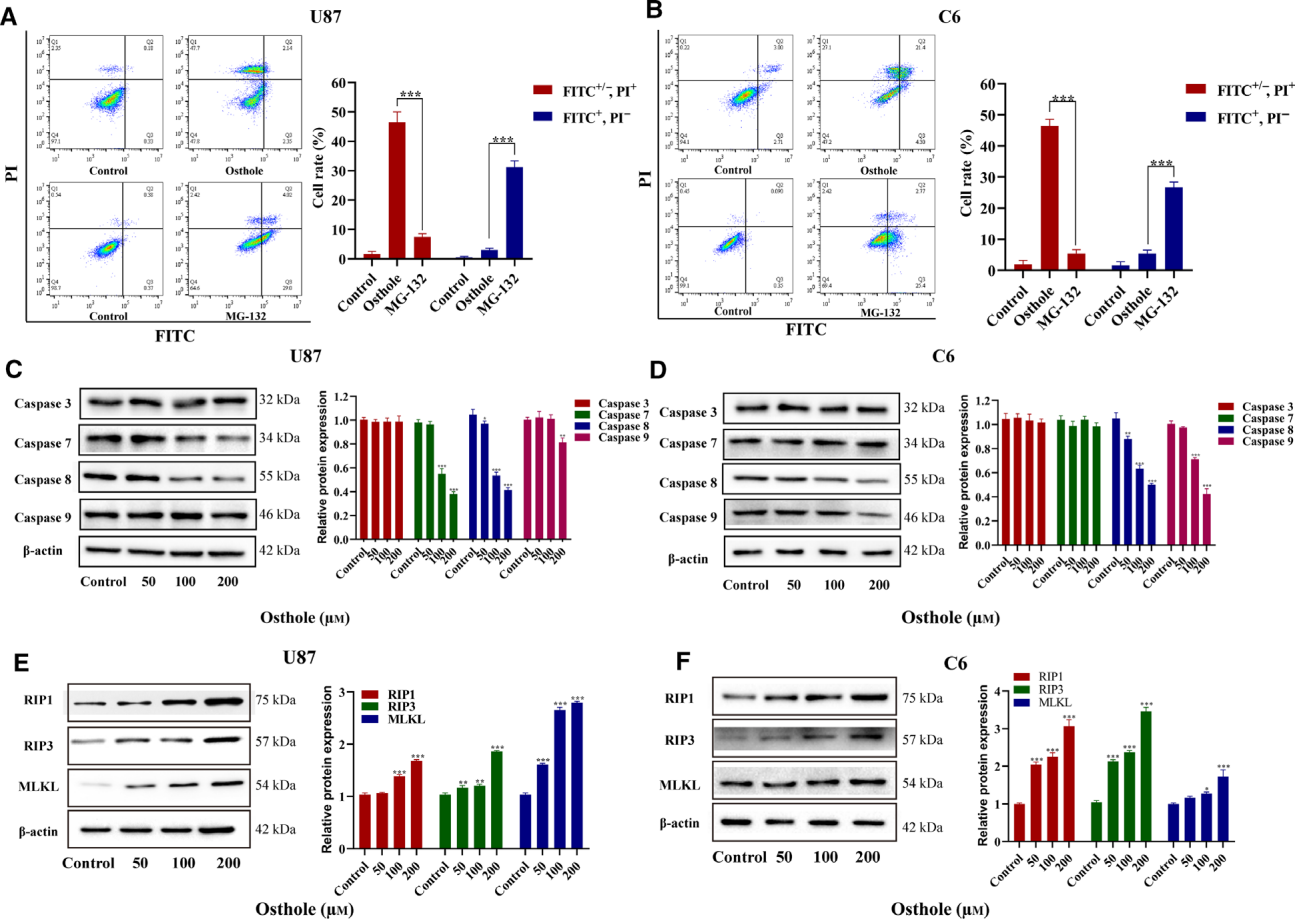
Osthole induced necroptosis in glioma cells. (A, B) U87 and C6 cells were treated with 200 μm osthole or 20 μm MG‐132 for 18 h and stained by Annexin Ⅴ–FITC/PI. The rate of different forms of cell death was quantified. (C, D) After treatment with indicated concentrations of osthole for 18 h, the protein expressions of caspase‐3, ‐7, ‐8, and ‐9 were detected by western blotting. The expression was quantified by image j, and β‐actin was used as a control. (E, F) The expressions of RIP1, RIP3, and MLKL were detected by western blotting. The expression was quantified by image j, and β‐actin was used as a control. Data were presented as mean ± SD (*n* = 3). ANOVA with Bonferroni's *post hoc* test was used to test differences between multiple groups, and Student's *t*‐test was used to test differences between two groups. *P < 0.05, **P < 0.01, ***P < 0.001.

To further clarify the mechanism of osthole‐induced glioma cell death, we analyzed the form of cell death induced by osthole and the typical apoptosis activator MG‐132. Annexin Ⅴ–FITC/PI double staining showed that almost only early apoptosis (FITC^+^, PI^+^) existed in MG‐132 treated cell death, whereas in osthole‐treated U87 and C6 cells, almost all dead cell were stained by PI (Fig. 2A,B). All these results suggest that the form of osthole‐induced glioma cells death was different from MG‐132.

Furthermore, we found that osthole treatment had no effect on caspase‐3 in U87 cells and on caspase‐3 and capase‐7 in C6 cells. The protein expressions of caspase‐7 and caspase‐9 in U87 cells and caspase‐9 in C6 cells were even decreased compared with the control group. Moreover, apoptosis‐related caspase‐8 was significantly decreased in both U87 and C6 cells (Fig. 2C,D), which indicated that osthole induced glioma cells necroptosis. Meanwhile, the cell distribution in C6 cells was similar to typical necroptosis induced by shikonin (Fig. 2B) [8, 10]. The key regulators of necroptosis, RIP1, RIP3 and MLKL, were up‐regulated in osthole‐treated cells (Fig. 2E,F). Therefore, we speculated that osthole induced necroptosis, but not apoptosis, in U87 and C6 cells.

### Nec‐1 attenuated osthole‐induced necroptosis in glioma cells

**Fig. 3:**
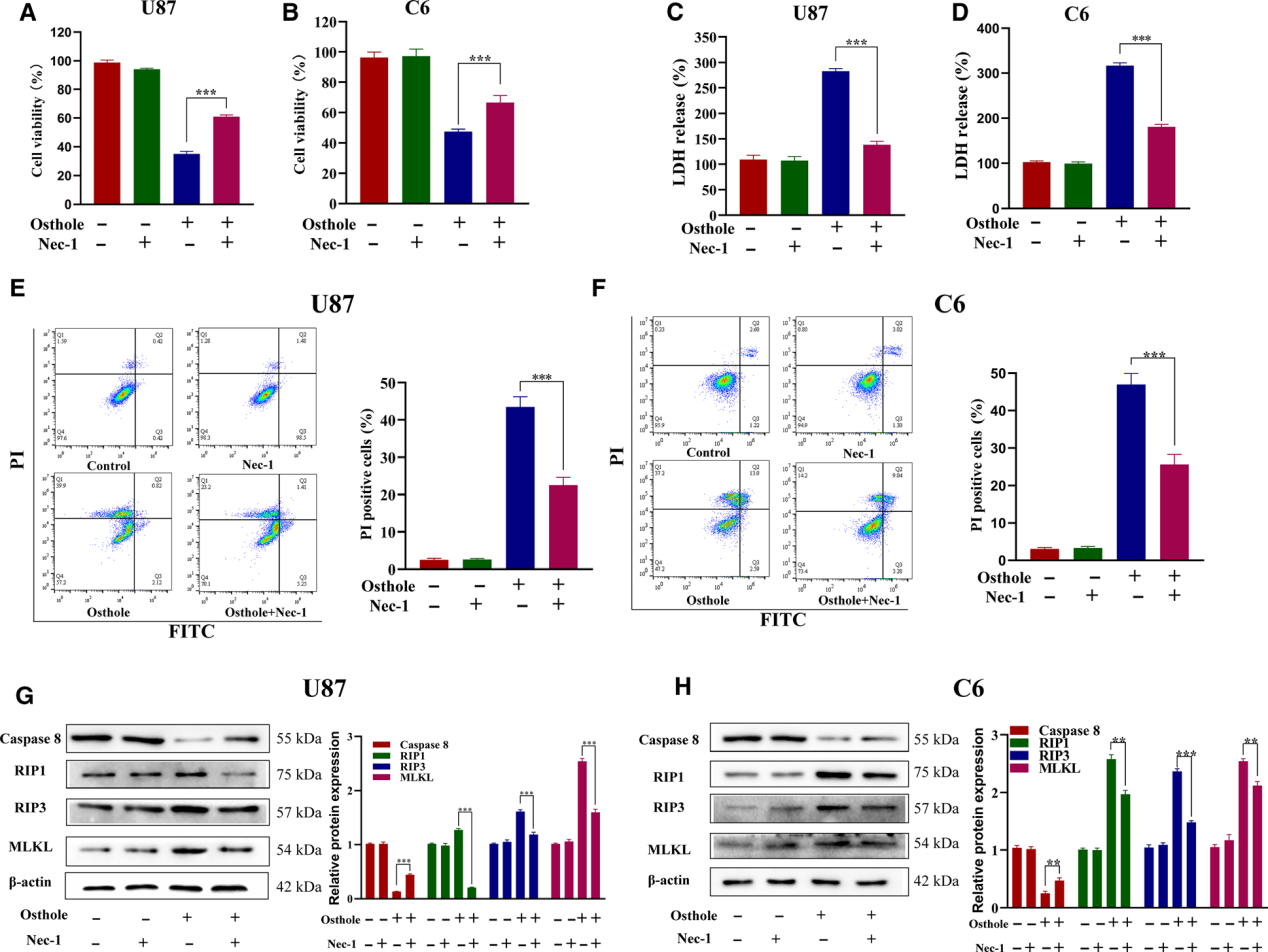
Osthole‐induced necroptosis was reversed by RIP1 inhibitor Nec‐1 in glioma cells. (A–H) U87 and C6 cells were treated with 200 μm osthole in the presence or absence of 100 μm Nec‐1 for 18 h, and the untreated cells were used as control. (A, B) Cell viability was detected by MTT assay. (C, D) LDH release was detected by LDH assay kit. (E, F) Annexin Ⅴ–FITC/PI staining was detected by flow cytometry. When pretreated with Nec‐1, the PI ^+^ cells induced by osthole decreased significantly. (G, H) The protein expressions of caspase‐8, RIP1, RIP3 and MLKL were detected by western blotting. The expression was quantified by image j, and β‐actin was used as a control. Data were presented as mean ± SD (*n* = 3). Student's *t*‐test was used to test differences between two groups. **P < 0.01, ***P < 0.001.

To explore the role that RIP1 played in osthole‐caused necroptosis, we pretreated cells with Nec‐1 for 1 h and treated with osthole for 18 h. MTT assay showed that pretreatment with Nec‐1 reversed osthole‐caused inhibition in cell viability (Fig. 3A,B). In addition, LDH release assay showed that Nec‐1 significantly lowered the osthole‐induced LDH release in glioma cells (Fig. 3C,D). Moreover, Annexin Ⅴ–FITC/PI staining results showed that pretreatment with Nec‐1 reduced cell necrosis caused by osthole in U87 and C6 cells. The histogram illustrated that Nec‐1 reduced PI uptake, which indicated that cell membrane integrity rupture caused by osthole was protected by Nec‐1(Fig. 3E,F). Western blotting showed that the decreased expression of caspase‐8 and the increased expression of RIP1, RIP3 and MLKL caused by osthole were reversed by Nec‐1 (Fig. 3G,H). These results suggested that RIP1 inhibitor reversed necroptosis induced by osthole.

### Osthole induced mitochondrial depolarization and ROS overproduction in glioma cells

**Fig. 4:**
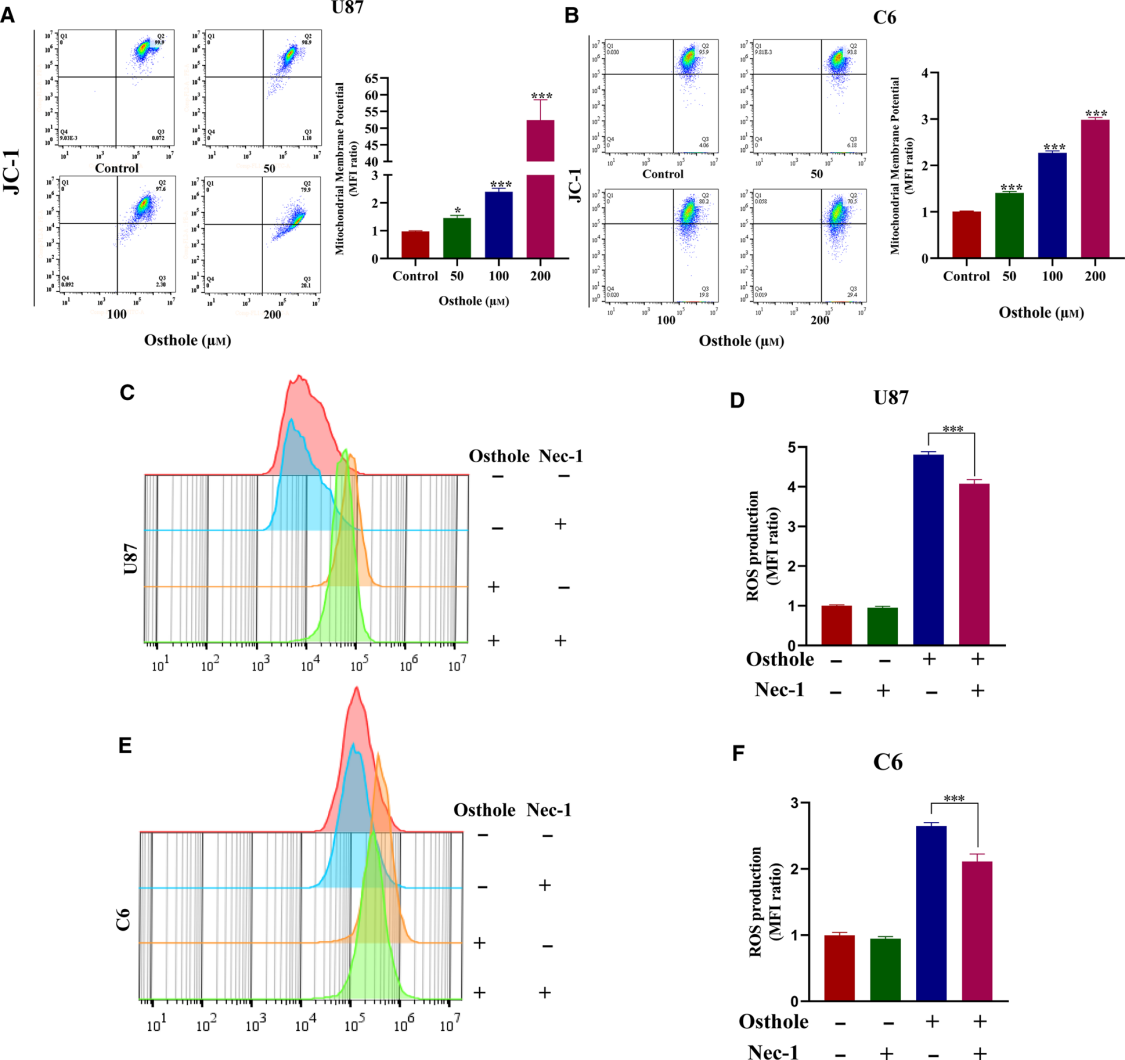
Osthole induced loss of MMP and ROS overproduction. (A, B) After treatment with osthole for 18 h, the JC‐1 staining was detected by flow cytometry in U87 and C6 cells. Quantification of JC‐1 MFI (green/red ratio) indicated the changes in MMP. (C, E) U87 and C6 cells were treated with 200 μm osthole in the presence or absence of 100 μm Nec‐1 for 18 h, and the untreated cells were used as control. The ROS production was detected by DCFH‐DA staining. (D, F) Quantification of DCFH‐DA MFI indicated the changes in ROS production. Data were presented as mean ± SD (*n* = 3). ANOVA with Bonferroni's *post hoc* test was used to test differences between multiple groups, and Student's *t*‐test was used to test differences between two groups. *P < 0.05, ***P < 0.001.

It has been reported that occurrence of necroptosis was often accompanied by decrease of MMP and overproduction of ROS [24, 25]. Thus, we detected the MMP in osthole‐treated cells by JC‐1 staining. JC‐1 is a fluorescent dye that presents red fluorescence at normal cells and transforms to green fluorescence at dying cells with loss of MMP. We found that osthole decreased red fluorescence and increased green fluorescence compared with the control group, which indicated mitochondrial depolarization. The quantification of MFI demonstrated that the green and red fluorescence in osthole‐treated cells was increased in a dose‐dependent manner (Fig. 4A,B). Then we determined ROS production by DCFH‐DA dye. The result demonstrated that pretreatment with Nec‐1 reduced the production of ROS compared with osthole‐treated U87 and C6 cells (Fig. 4C,E). Quantification of MFI offered further evidence to prove the reversal effect of Nec‐1 on ROS overproduction induced by osthole (Fig. 4D,F). In summary, osthole induced necroptosis, as well as mitochondrial depolarization and ROS production, in glioma cells.

### ROS contributed to necroptosis in glioma cells

**Fig. 5:**
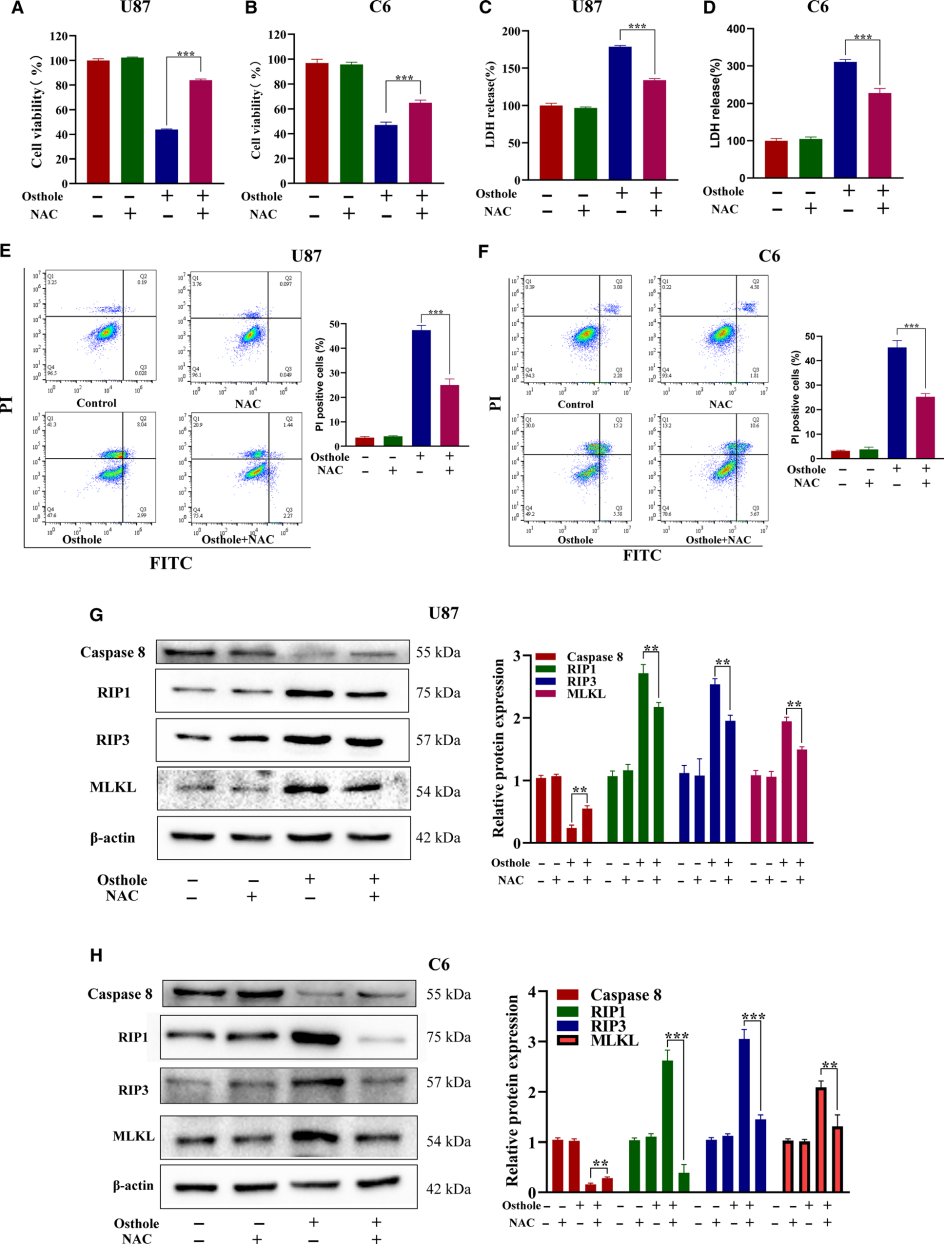
ROS contributed to osthole‐induced necroptosis in glioma cells. (A‐H) U87 and C6 cells were treated with 200 μm osthole in the presence or absence of 2 μm NAC for 18 h, and the untreated cells were used as control. (A, B) Cell viability was detected by MTT assay. (C, D) LDH release was detected by LDH assay kit. (E, F) Annexin Ⅴ–FITC/PI staining was detected by flow cytometry. When pretreated with NAC, the PI^+^ cells induced by osthole decreased significantly. (G, H) The protein expressions of caspase‐8, RIP1, RIP3 and MLKL were detected by western blotting. The expression was quantified by image j, and β‐actin was used as control. Data were presented as the mean ± SD (*n* = 3). ANOVA with Bonferroni's *post hoc* test was used to test differences between multiple groups, and Student's *t*‐test was used to test differences between two groups. **P < 0.01, ***P < 0.001.

Because ROS was overproduced in osthole‐treated glioma cells, we wonder whether it was related to the occurrence of necroptosis. We first pretreated cells with ROS inhibitor NAC and found that the decreased cell viability caused by osthole was turned back (Fig. 5A,B). In addition to cell viability, LDH release was also reserved by NAC (Fig. 5C,D). Annexin Ⅴ–FITC/PI double‐staining results showed that NAC abrogated osthole‐induced cell death in U87 and C6 cells, and PI uptake was also reversed by NAC (Fig. 5E,F). Moreover, the expressions of caspase‐8, RIP1, RIP3 and MLKL were reversed by NAC in U87 and C6 cells (Fig. 5G,H), which intimated that osthole‐induced necroptosis was regulated by ROS. Interesting is that osthole seemed to induce a positive feedback loop between ROS and RIP1.

## Discussion

PCD, primarily known as apoptosis, recently attracted more researchers’ attention for its newly found cell death form, such as necroptosis [11], ferroptosis [26] and parthanatos [27]. As a new form of PCD, necroptosis provides new insights into the treatment of drug resistance cancers [28]. A few natural compounds, such as shikonin [10, 29], 2‐methoxy‐6‐acetyl‐7‐methyljuglone [25] and bufalin [9], can trigger necroptosis in cancer cells. As for osthole, a natural coumarin, previous reports were focused on its apoptosis induction in cancer cells [20, 30]. The mechanisms of osthole‐induced cell apoptosis were via Bax‐ and Bcl‐2‐related mitochondrial apoptotic pathways [18, 23, 31] and the phosphatidylinositol 3 kinase (PI3K)/Akt pathway [30, 32]. In addition, osthole could induce caspase‐3‐mediated apoptosis and then cleave gasdermin E N‐terminal domain to induce pyroptosis [33]. All these death mechanisms were associated with apoptosis, and there was no relevant report about its antitumor effect of triggering necroptosis. In this study, we found that osthole inhibited the viability of U87 and C6 cells, which was accompanied by LDH release and PI uptake. The change of plasma membrane is one of the ways to distinguish the manner of necrosis [34]. LDH release [35] and PI uptake [36] were generally considered a sign of membrane integrity loss. In this study, we found that osthole increased LDH release in a dose‐dependent manner, and Annexin Ⅴ–FITC/PI staining showed that cells were concentrated in a PI‐positive group. These findings supported that osthole induced plasma membrane rupture and necrosis in glioma cells.

Previous reports have confirmed that RIP1, RIP3 and MLKL are crucial biomarkers of necroptosis [36, 37], which will be inhibited by the apoptosis inducer caspase‐8 when necroptosis happened and prevents the formation of necrosome [12]. In this study, we found that osthole up‐regulated the expression of RIP1, RIP3 and MLKL and significantly down‐regulated the expression of caspase‐8. These results suggested that osthole induced necroptosis in glioma cells. Pretreated with RIP1 inhibitor, Nec‐1 could significantly reverse osthole's effect on necroptosis induction and changes of caspase‐8, RIP1, RIP3 and MLKL expressions. Thus, our results suggested that osthole activated necroptosis via the RIP1/RIP3/MLKL signaling pathway in glioma U87 cells.

Even though the ROS level in cancer cells is higher than in normal cells, ROS production at an abnormally high level can trigger various cell deaths [16]. The occurrence of necroptosis is usually accompanied by an overproduction of ROS [38]. ROS production plays a crucial role in many drug‐induced necroptosis cases [39, 40]. Simultaneously, the generation of ROS is frequently accompanied by mitochondrial damage and reduction of MMP. However, the mechanism of ROS in necroptosis has not been further elucidated. As the initiation factor of necroptosis, RIP1 plays an important role in necroptosis, and it has been reported to contribute to ROS production [9]. In this study, we found that RIP1 inhibitor Nec‐1 inhibited the overproduction of ROS caused by osthole, whereas ROS inhibitor NAC also reduced the up‐regulated expression of RIP1, RIP3 and MLKL induced by osthole in U87 and C6 cells, which indicates that ROS plays an important role in the regulation of osthole‐mediated glioma cell necroptosis, and osthole may induce a positive feedback loop between ROS and RIP1.

In summary, osthole was identified as a necroptosis trigger in human glioma cells. The occurrence of necroptosis mediated by osthole was mainly through the ROS‐mediated RIP1/RIP3/MLKL pathway. Our study provided new insights into the anticancer mechanisms of osthole, and osthole may be a potential alternative drug for glioma therapy.

## Conflict of interest

The authors declare no conflict of interest.

## Author contributions

MH, RW and XC participated in the design of the experiment. MH, JQ, DY, XG and XL performed the experiments. JW wrote the paper and revised the manuscript. MF and HL were involved in data analysis and picture layout. All authors approved the final manuscript.

## Supporting information


**Fig. S1.** Osthole induced increased LDH release in U87 and C6 cells with the increased incubation time. (A, B) LDH release in U87 and C6 cells was tested after treatment with 200 μm osthole for 12, 18 and 24 h. Data were presented as the mean ± SD (*n* = 3). ANOVA with Bonferroni's *post hoc* test was used to test differences between multiple groups, and Student's *t*‐test was used to test differences between two groups. **P < 0.01, ***P < 0.001, ^NS^ *P > *0.05.

## Data Availability

The data will be available from the corresponding author on reasonable request.
