# Supplementary figures and images for: Osthole induces necroptosis via ROS overproduction in glioma cells
Source: FEBS Open Bio. 2021 Jan 19;11(2):456–67. doi: 10.1002/2211-5463.13069 (PMC7876487; doi:10.1002/2211-5463.13069)

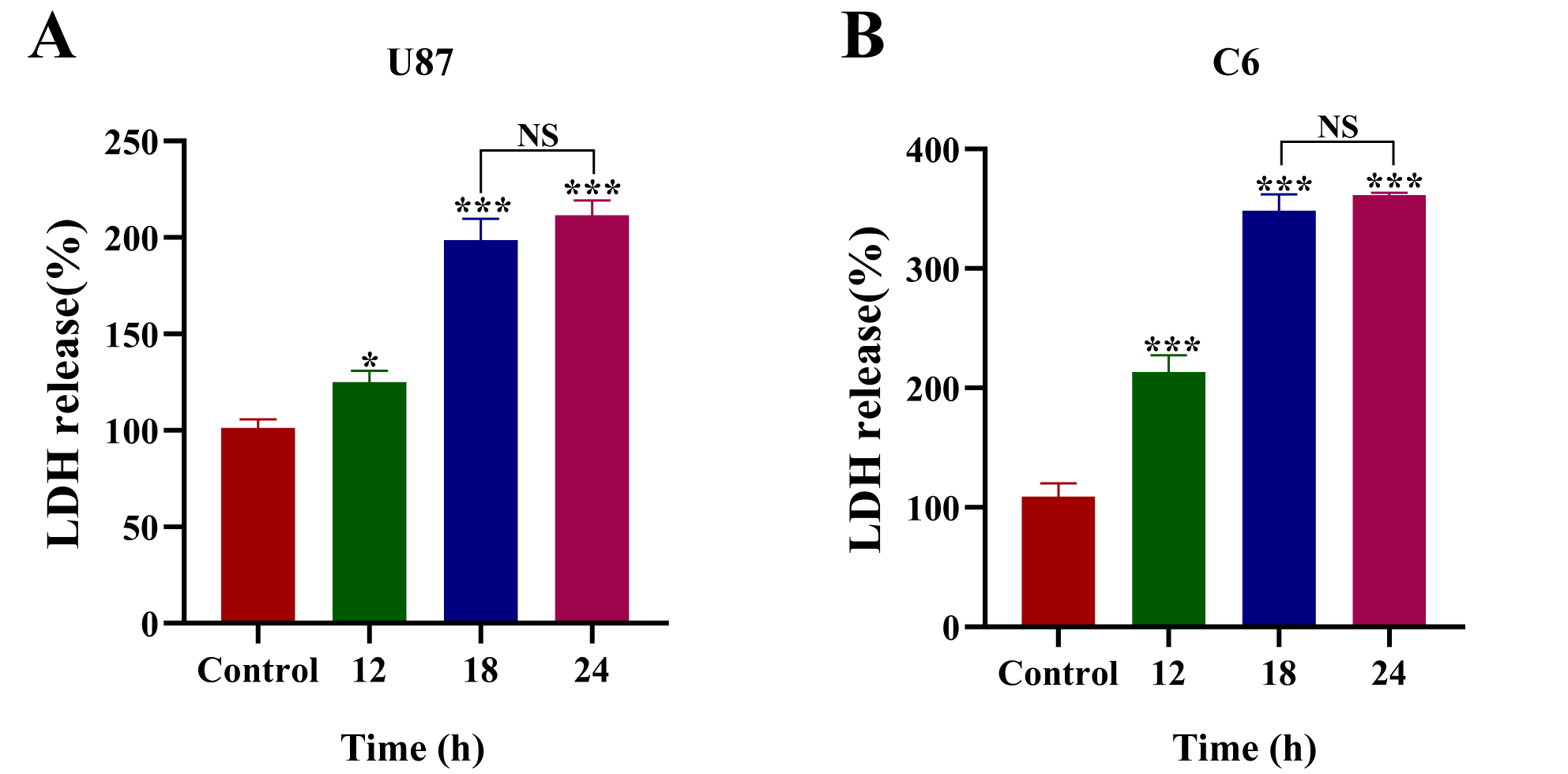

Supplement: Supplementary file 1 — Fig. S1. Osthole induced increased LDH release in U87 and C6 cells with the increased incubation time. (A, B) LDH release in U87 and C6 cells was tested after treatment with 200 μm osthole for 12, 18 and 24 h. Data were presented as the mean ± SD (n = 3). ANOVA with Bonferroni's post hoc test was used to test differences between multiple groups, and Student's t‐test was used to test differences between two groups. **P < 0.01, ***P < 0.001, NS P > 0.05. [file FEB4-11-456-s001.tif]
